# Does adolescent depression modify the association between psychosocial job stressors and mental health in emergent adulthood?

**DOI:** 10.1002/ajim.23547

**Published:** 2023-11-04

**Authors:** Anthony D. LaMontagne, Lay‐San Too, Katrina Witt, Tracy Evans‐Whipp, Patrick J. Owen, John W. Toumbourou

**Affiliations:** ^1^ Institute for Health Transformation Deakin University Geelong Victoria Australia; ^2^ Melbourne School of Global & Population Health University of Melbourne Melbourne Victoria Australia; ^3^ Centre for Youth Mental Health The University of Melbourne Melbourne Victoria Australia; ^4^ Orygen Parkville Victoria Australia; ^5^ Australian Institute of Family Studies Southbank Victoria Australia; ^6^ Department of Paediatrics The University of Melbourne Melbourne Victoria Australia; ^7^ Centre for Social and Early Emotional Development (SEED) Deakin University Geelong Victoria Australia

**Keywords:** depression, depression symptoms, emergent adults, job stressors, Kessler‐10, psychosocial work environment

## Abstract

**Background:**

Job stressors can be particularly harmful to the mental health of disadvantaged groups through differential exposure, differential sensitivity to the effects of exposure, or both. In this paper, we assess the extent to which emergent adult workers with an adolescent history of high depression symptoms may be differentially sensitive to the effect of job stressors on mental health.

**Methods:**

We conducted a secondary analysis of three waves of the Australian arm of the International Youth Development Study (*n* = 1262). We used multivariable linear regression to assess whether self‐reported measures of high depression symptoms at one or two time points in adolescence (ages 11–16 years) modified the cross‐sectional association between four self‐reported job stressors (job demands, job control, job strain, and incivility at work) and psychological distress (Kessler‐10 scores) in emergent adulthood (ages 23–27 years).

**Results:**

For all four job stressors, there was a consistent pattern of approximately a doubling in the magnitude of association for participants with a history of high depression symptoms at two points in adolescence compared with those with no history of depression. However, results of effect modification analysisfor only job demands and job strain excluded chance as a potential explanation.

**Conclusions:**

Findings showed partial support for the hypothesis that a history of high depression symptoms in adolescence predicts stronger associations between job stressor exposures and psychological distress among those employed in emergent adulthood. The limitations of this secondary analysis suggest a need for purpose‐designed studies to answer this important research question more definitively.

**Abbreviations**
IYDS: International Youth Development Study
K10: Kessler‐10
SMFQ: Short Moods and Feelings Questionnaire
T1, T2, T3: time 1, time 2, time 3

## 1 BACKGROUND

There is growing interest in understanding the determinants of mental health in young people in the life stage of “emergent adulthood”: people aged 18–29 years.^1^ The age of onset of many mental disorders is in adolescence and emergent adulthood, and this is also the period of highest prevalence for many disorders. In the United States, for example, three‐fourths of anxiety, mood, impulse control, and substance abuse disorders have their onset by age 24, and the 1‐year prevalence of any psychiatric disorder is more than 40% in the emergent adulthood period.^2^ In Australia, 16–25‐year‐olds have the highest prevalence of mental illness of all age groups.^3^

Obtaining gainful employment is one of the essential life challenges of emergent adulthood. In today's labor market, employment experiences in emergent adulthood tend to be characterized by frequent job changes, and periods of unemployment and underemployment, each of which can adversely influence mental health.^4^, ^5^, ^6^, ^7^ Further, these employment transitions have increased in frequency during the COVID‐19 pandemic in Australia and elsewhere.^8^ While employed, psychosocial job quality—defined as combined exposures to job stressors such as psychologic demands, job control and job insecurity—is also an important determinant of mental health in this period. Emergent adult workers, for example, are disproportionately exposed to low job control and high job insecurity,^9^ which are established risk factors for adverse mental health outcomes.^10^, ^11^, ^12^ On the other hand, it has been shown that young people transitioning into jobs with high psychosocial job quality experience improved mental health while transitioning into poor psychosocial quality jobs can be as harmful to mental health as unemployment.^13^ In short, work and working conditions are prominent modifiable determinants of mental health for emergent adults, and work can influence mental health both for better and for worse.

Psychosocial job quality may be particularly important for emergent adults with a history of mental health problems. Given the high prevalence of mental disorders among young people,^2^, ^3^ this may affect a substantial proportion of the emergent adult workforce. Globally, the WHO estimates that 14% of 10–19‐year‐olds experience mental health conditions.^14^ It has been previously shown that emergent adults with a history of mental health problems are less likely than their peers to have completed secondary education, and less likely to be in paid employment in the emergent adulthood period, including in Australia.^15^ In absolute terms, however, most young people with a history of mental health problems, however, do find employment in emergent adulthood. Across OECD countries (industrialized democracies), an estimated 60% of people with common mental health problems are working.^16^ In an Australian study based on the same cohort being analyzed in this paper, roughly three‐quarters of emergent adults with an adolescent history of depression symptoms were employed in their late 20's.^15^

There is an acknowledged dearth of studies on the impact of mental health problems in adolescence on the experience of work in adulthood.^17^ A recent study showed that a history of childhood or adolescent mental health problems impacted the work functioning workers of young adult workers, in particular experiencing difficulties in meeting their work demands.^18^ Other research suggests that people with a history of mental health problems tend to be employed in poor psychosocial quality jobs (e.g., low job control, high job insecurity, higher prevalence of bullying) compared with those without such a history.^19^ Or, stated another way, people with a history of mental health problems might tend to be differentially exposed to poor psychosocial working conditions.^20^, ^21^ This would be a concern for the sustainability of employment for these workers as exposure to poor psychosocial working conditions could exacerbate their mental health problems or contribute to recurrence.^10^, ^11^, ^12^ Differential exposure could arise due to selection into poorer psychosocial quality jobs arising from their work limitations, lower educational attainment, or other factors associated with their illness history. A large French working population study found this to be the case; in this study, a history of adolescent‐onset mental illness was associated with elevated exposures to low social support at work, role conflict, job insecurity, and night work compared with workers under 30 years of age without a history of mental illness.^22^ In contrast, an Australian population‐based study—based on the same cohort being analyzed in this paper—found that a history of adolescent depression predicted higher exposure to workplace incivility (bullying), but not low control, high demands, or job strain in their late 20's.^15^ While these differences may in part stem from factors associated with geographical location and associated differences in employment conditions (e.g., access to workplace mental health services) and policy (e.g., OH&S regulatory regimes), differences in sample demographics should also be considered. For example, poor socioeconomic status (SES) in childhood was greater in the Australian cohort (21.6% vs. 7.7%), whereas current employment was greater in the French cohort (89.8% vs. 72.5%).

In describing “differential exposure,” it is important to acknowledge that self‐reports of job stressors are, strictly speaking, measures of perceptions, not objective working conditions. The weight of the evidence, however, supports a strong relationship between worker perceptions and objectively measured job stressors; for example, self‐reports have been validated against objective measures based on job‐exposure matrices, group assignment of exposures (e.g., by occupational group), and observer‐based measures.^23^, ^24^ Hence, we argue that self‐report measures do reasonably represent job stressor exposures.

Differential exposure to poor psychosocial job quality may also be further compounded by differential sensitivity or susceptibility to the impacts of such exposures on mental health.^20^, ^21^ There is indeed precedent for the phenomenon of differential sensitivity in this area. For example, effort‐reward imbalance and job strain were substantially more strongly related to depression among lower occupational status workers compared with higher status workers.^25^ Other forms of disadvantage may also confer higher sensitivity or susceptibility to job stressors, such as migrant or racial minority status.^20^ In the present context, adolescent experience of depression could modify the association between job stressors and mental health by eroding self‐esteem and mastery, which have been proposed as potential mechanisms linking job stressor exposures to mental health^26^; a history of depression could either create a lower threshold of job stressor exposure, or an increased sensitivity to job stressor exposures, in relation to adverse mental health impacts.

The present study aims to contribute to addressing these research gaps for the purpose of informing strategies for sustaining employment among young workers with a history of mental health problems.^17^ In this paper, we assess the extent to which emergent adults with a history high depression symptoms (as a proxy for depression caseness) are differentially sensitive to job stressors in relation to a scaled measure of mental health. Utilizing three waves of longitudinal data from the International Youth Development Study (IYDS),^27^ we conducted a secondary analysis to assess the extent to which high depression symptoms at one or both time points in early adolescence modified the association between job stressors and Kessler‐10 (K10) scores in emergent adulthood. We hypothesize that there will be a stronger association between job stressor exposures and K10 scores in emergent adulthood among those who experienced high depression symptoms in adolescence compared with those without such a history. If confirmed, this would have implications for workplace mental health policy and practice, including for the design of supported employment strategies for young workers.

## 2 METHODS

### 2.1 Participants

This study used data from the Australian arm of the IYDS. Full details of IYDS sampling, recruitment, and procedure are described elsewhere.^27^ In brief, a two‐stage random sampling approach was employed whereby the first stage selected schools within Victoria, Australia, and the second selected classrooms within these schools. Collectively, 65% (165/254) eligible classrooms agreed to participate. Schools identified tended to proportionately represent school type (government/public, independent, Catholic) and student diversity (based on primary language spoken at home), yet slightly overrepresented student families receiving low‐income assistance. Among eligible students, parental consent was obtained from 73.5% (2884/3926), with the corresponding children providing assent to complete the survey. Ethics approval for the original IYDS study was provided by the [REDACTED] Hospital Ethics in Human Research Committee in Victoria (Australia). Ethics approval for the analysis presented in this paper was granted by [REDACTED] University's Human Research Ethics Committee, protocol #2017‐124. Written informed consent was obtained from all subjects and/or their legal guardian(s), and the study was carried out in accordance with the Declaration of Helsinki.

In the present analysis, data were collected at Wave 1 in 2002, Wave 2 in 2003, and Follow‐up 3 roughly a decade later in 2014 (where measures of job stressors were introduced). At Wave 1 (T1), respondents were between 11.2 and 14.2 years of age; at Wave 2 (T2), respondents were between 12.2 and 15.6 years; and finally, at Follow‐Up 3 (T3), respondents were between 23.3 and 26.6 years. Retention rates at 1‐year follow‐up (T2) in 2003 were 99%, and at 12‐year follow‐up (T3) in 2014 were 86%. We excluded those who were classified “No” to a measure of “honesty” (*n* = 38); this measure was computed based on student responses to three survey items, including use of a fictional drug.^27^ Our analysis was restricted to respondents who were employed at T3 and who had responded to relevant variables in all waves (complete cases). Supporting Information: Figure 1 presents a flow chart detailing the specification of the analytic sample.

### 2.2 Outcome variable

Psychological distress at T3, when participants were aged 24‒29 years, was assessed using the 10‐item Kessler Psychological Distress Scale.^28^ The K10 was based on questions about anxiety and depressive symptoms that an individual has experienced in the last 4 weeks. It used a 5‐point Likert scale, ranging from 1 = *none of the time* to 5 = *all of the time*. We summed the scores of all items for each respondent to approximate their psychological well‐being, with a higher score indicating a greater level of psychological distress (possible range 10–50). Higher K‐10 scores reflect adverse psychological symptoms associated with mood fluctuations and correlate with common mental disorders.

### 2.3 Exposure variables

#### 2.3.1 Job control and job demands

Job exposures were based on data at T3. Job control and job demands were assessed using 11 items from the Karasek job content questionnaire.^29^, ^30^ Each item offered four response categories: “strongly agree,” “agree,” “disagree,” and “strongly disagree.” Eight items assessed job control (e.g., skill discretion: “My job requires that I learn new things”; decision authority: “My job allows me to make a lot of decisions on my own”), which was composed of an equally weighted sum of skill discretion and decision authority (Cronbach's *⍺* = 0.90). Three items assessed job demands (e.g., “I am not asked to do an excessive amount of work”) (Cronbach's *⍺* = 0.76). For each job measure, the sums of item scores were calculated and dichotomized into “high” and “low” using their median scores, with scores above the median indicative of adverse job conditions (low job control, high job demands).^24^ By using this approach, we specified a smaller proportion of respondents with adverse job conditions than that of respondents without the adverse job condition.

#### 2.3.2 Job strain

High job strain was defined as the combination of low job control and high job demands, while the other three combinations were categorized collectively as low job strain.

#### 2.3.3 Workplace incivility

Workplace incivility in the past 6 months was based at T3 and was based on three items (“In the past 6 months, how often have you been ignored or excluded at work?,” “How often have you been humiliated or ridiculed at work?,” and “How often has someone withheld information that affected your performance?”).^31^ Each item contained six response categories: “never,” “now and then,” “monthly,” “weekly,” and “daily.” Respondents who indicated that they experienced any of these incivilities in the past 6 months were grouped into “Yes” (1) and those who did not were grouped into “No” (0). The reliability for job incivility measure used was high (Cronbach's *α* = 0.89).

### 2.4 Effect modifier

The Short Moods and Feelings Questionnaire (SMFQ)^32^ was used to assess high depression symptoms or depression “caseness” in adolescence. The SMFQ comprises 13 items (e.g., “I felt miserable or unhappy” and “I felt so tired I just sat around and did nothing”) that assessed participants' mood, feelings, and behaviors in the past 30 days, with three response options (2 = *true*, 1 = *sometimes true*, and 0 = *not true*). The sum score of these items was computed and dichotomized into two categories. Consistent with other work using data from the IYDS, a score of ≥11 was used to indicate diagnosable depression in this study.^33^ Cronbach's *α* for the SMFQ was 0.86 for T1 and 0.91 for T2.^34^ The SMFQ has good criterion validity when assessed against the Diagnostic Interview Schedule for Children depression scale,^32^, ^35^ the International Classification of Disease—Version 10 (ICD‐10), and the Diagnostic Statistical Manual‐Fourth Revision (DSM‐IV),^36^ and has previously been used to indicate depression “caseness.”^33^, ^37^ We constructed an effect modifier variable as a measure of history of high depression symptoms (depression caseness). This variable was coded as 0 = *neither at T1 nor T2*; 1 = *yes at either T1 or T2, but not both*; and 2 = *yes at both T1 and T2*.

### 2.5 Covariates/potential confounders

We adjusted for measured variables that could potentially confound the relationship between job stressors and mental health.^11^, ^23^ These included gender and family SES in adolescence as well as partner status, and educational attainment in adulthood. (also a possible mediator). Gender was measured by self‐report at T1 and included two response options: male or female. A single composite measure of family SES, also measured at T1, was formed based on parents' responses to questions on their education status (“What your highest level of education?”) and family income (“What is your family's combined yearly income before taxes?”). This was an ordinal measure ranging from 1 to 3, where a higher score indicated a higher level of SES.

For partner status, measured at T3, those who indicated that they were engaged, married, or living with partner were categorized as “having a partner” while those indicating they were single, not married, divorced, separated, or widowed were categorized as “not having a partner.”

A composite measure of educational attainment, measured at T3, was generated based on two questions: “What was the highest year level at secondary school you completed?” and “What is the highest level of education you have completed, since secondary school?” We classified this variable into four categories: tertiary qualification (postgraduate/bachelor's degree, diploma/certificate), Year 12, and Year 11 or below.

We also acknowledge that in addition to potentially operating as an effect modifier of the job stressor‐K10 relationship, history of high depression symptoms may also be a confounder (Supporting Information: Figure 1), and therefore, we also considered the potential confounding role of this variable.

### 2.6 Statistical analysis

All analyses were performed using Stata (v17, StataCorp). To assess the potential for bias in the sample, key variable frequencies or means were compared between the 141 excluded observations from the 1401 participants working versus the 1262 included participants with complete data. We reported descriptive statistics of the characteristics of respondents at T1 and descriptive statistics of exposures and outcome at T3, and hypothesized effect modifier at T1 and T2. We used simple linear regression models to estimate the relationship between job stressors and K10 score in adulthood at T3, including adjustment for gender at T3, family SES at T1, and partner status at T3. The potential for effect modification of the job stressor‐K10 relationship in adulthood by adolescent experience of depression was examined by Wald test of the interaction and comparison of the difference in estimated marginal effects emanating from the model including the product term (dichotomous exposure*three‐category effect modifier), providing a measure of the magnitude of relative excess risk of interaction on an additive scale.^38^ Effect modification was deemed likely when both the interaction and at least one difference in estimated marginal effects was statistically significant. Finally, given the potential relationships between job stressors in the current study, a supplemental analysis used multiple linear regression models to estimate the relationship between mutually adjusted job stressors and K10 scores in adulthood at T3, including adjustment for adolescent history of high depression symptomology, gender at T3, family SES at T1 and partner status at T3. Further, analyses were conducted with and without including educational attainment at T3 as a potential confounder because it could also be on the causal pathway between history of depression and differential sensitivity to job stressors in adulthood; coefficients were unappreciably affected, so educational attainment was not included in final models (data not shown). Further, job stressor coefficients were attenuated but remained significantly associated with K10 scores with mutual adjustment (Supporting Information: Table 3), so effect modification analyses were conducted for job stressors separately to conserve statistical power.

An *α* of 0.05 was adopted for all analyses.

## 3 RESULTS

There were no significant differences in the distributions or means of covariates, exposures, the effect modifier, and K10 outcomes between the ~10% of excluded versus included observations (Supporting Information: Table 1).

Demographic and other sample characteristics are presented in Table 1. At the third time point (T3, in emergent adulthood), there were slightly more females than males, roughly half were partnered, and most had completed secondary schooling (Year 12 in Australia). The vast majority of those who had completed secondary schooling had completed further education in the form of a diploma, certificate, or degree. Less than half of the respondents indicated they had experienced low job control, while around one‐third indicated they had experienced high job demands. Almost half of participants reported that they had experienced incivility at work over the previous 6 months. High job strain, the combination of low job control and high job demands, showed the lowest prevalence of the four job stressors.

**Table 1:** Sample characteristics (*n* = 1262).

Variable	Total sample
Gender at T3, *n* (%)	
Male	613 (48.6)
Female	649 (51.4)
Partner status at T3, *n* (%)	
Having a partner	645 (51.1)
Not having a partner	617 (48.9)
Education attainment at T3, *n* (%)	
Postgraduate/bachelor degree	600 (47.5)
Diploma/certificate	530 (42.0)
Year 12	97 (7.7)
Year 11 or below	35 (2.8)
Socioeconomic status at T1, mean (SD)	1.97 (0.50)
Job control at T3, *n* (%)	
High	761 (60.3)
Low	501 (39.7)
Job demand at T3, *n* (%)	
Low	836 (66.2)
High	426 (33.8)
Job strain at T3, *n* (%)	
Low	1,063 (84.2)
High	199 (15.8)
Incivility at work at T3, *n* (%)	
No	632 (50.1)
Yes	630 (49.9)
Psychological distress at T3, mean (SD)	18.72 (6.74)
History of high depression symptoms, *n* (%)	
Neither T1 nor T2	815 (64.6)
Either T1 or T2	279 (22.1)
Both T1 and T2	168 (13.3)

*Note*: Data are mean (SD) or count (%).

Abbreviations: T1, time 1; T2, time 2; T3, time 3.

With respect to our hypothesized effect modifiers, approximately two‐thirds were classified as not exceeding the SMFQ threshold for depression caseness (high depression symptoms) at both T1 or T2, with the remaining one‐third exceeding the threshold at either or both T1 and/or T2 (Table 1).

Each of the job stressors showed associations with K10 scores in the anticipated direction (i.e., adverse exposure associated with higher distress), but by varying magnitudes (Table 2, and Supporting Information: Table 2). Incivility at work was associated with K10 scores at nearly three times the magnitude of low job control, which showed the lowest magnitude of association with K10. Though history of high depression symptoms in adolescence strongly predicted K10 score at T3, adolescent history of depression as a potential confounder did not appreciably affect job stress‐K10 coefficients (at most a ~10% attenuation of coefficient for incivility), hence there was little evidence of adolescent history of depression operating as a confounder (Supporting Information: Table 2).

**Table 2:** History of high depressive symptomatology as a potential effect modifier of job stressor (job control, job demand, and incivility at work) and psychological distress (K10) relationships at T3 in adulthood.

Variable	Yes	No	*β* (95%CI)	*p* Value	Δ*β* (95% CI)	*p* Value
Low job control						
Neither T1 nor T2	336 (67.1)	479 (62.9)	1.03 (0.12, 1.93)	**0.026**		
Either T1 or T2	102 (20.4)	177 (23.3)	1.91 (0.34, 3.49)	**0.017**	0.88 (−0.94, 2.70)	0.341
Both T1 and T2	53 (12.6)	105 (13.8)	2.83 (0.81, 4.85)	**0.006**	1.80 (−0.41, 4.01)	0.110
High job demand						
Neither T1 nor T2	261 (61.3)	554 (66.3)	2.25 (1.30, 3.19)	**<0.001**		
Either T1 or T2	96 (22.5)	183 (21.9)	0.92 (−0.66, 2.50)	0.255	−1.33 (−3.17, 0.51)	0.157
Both T1 and T2	69 (16.2)	99 (11.8)	4.65 (2.68, 6.62)	**<0.001**	2.41 (0.22, 4.59)	**0.031**
High job strain						
Neither T1 nor T2	127 (63.8)	688 (64.7)	1.89 (0.68, 3.11)	**0.002**		
Either T1 or T2	41 (20.6)	238 (22.4)	2.21 (0.08, 4.34)	**0.042**	0.32 (−2.14, 2.77)	0.800
Both T1 and T2	31 (15.6)	137 (12.9)	5.31 (2.81, 7.82)	**<0.001**	3.42 (0.64, 6.20)	**0.016**
Incivility at work						
Neither T1 nor T2	374 (59.4)	441 (69.8)	2.98 (2.12, 3.84)	**<0.001**		
Either T1 or T2	152 (24.1)	127 (20.1)	4.08 (2.60, 5.55)	**<0.001**	1.10 (−0.61, 2.81)	0.208
Both T1 and T2	104 (16.5)	64 (10.1)	4.99 (3.05, 6.94)	**<0.001**	2.02 (−0.12, 4.15)	0.064

*Note*: Bold values indicate statistically signidicant at *p* > 0.05. Data are count (percentage), *β* (95% CI), and corresponding *p* value or difference (Δ) in *β* (95% CI) compared with reference of neither T1 nor T2. Analyses additionally adjusted for gender, partner status, and socioeconomic status.

Abbreviations: T1, time 1; T2, time 2; T3, time 3.x

**Figure 1:**
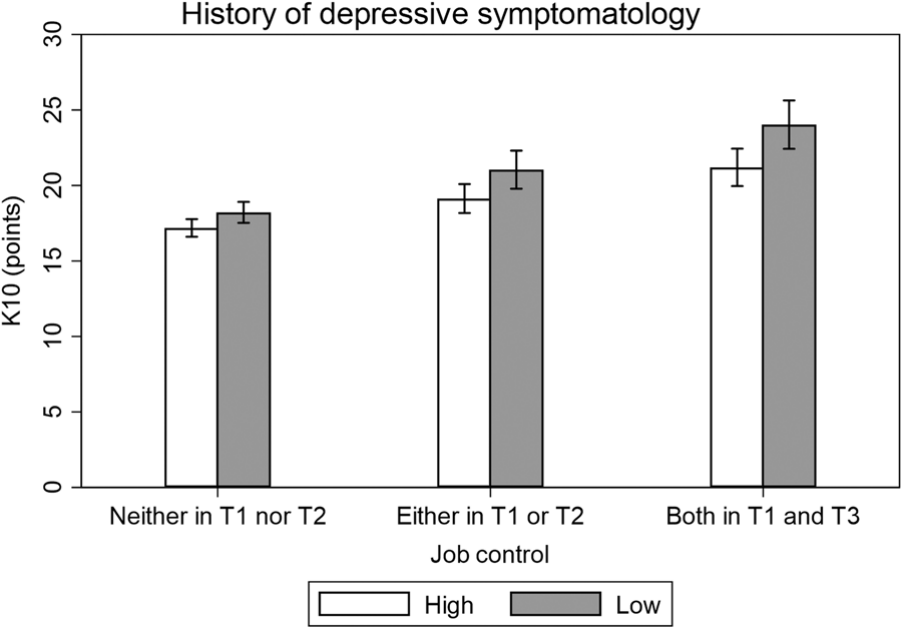
Estimated marginal mean (95% CI) effects of job control on psychological distress (K10) by history of depressive symptomatology. Data are adjusted for gender, partner status, educational attainment, and socioeconomic status.

**Figure 2:**
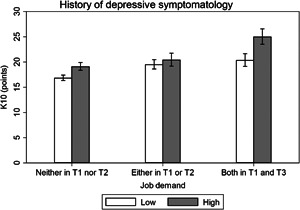
Estimated marginal mean (95% CI) effects of job demand on psychological distress (K10) by history of depressive symptomatology. Data are adjusted for gender, partner status, educational attainment, and socioeconomic status.

**Figure 3:**
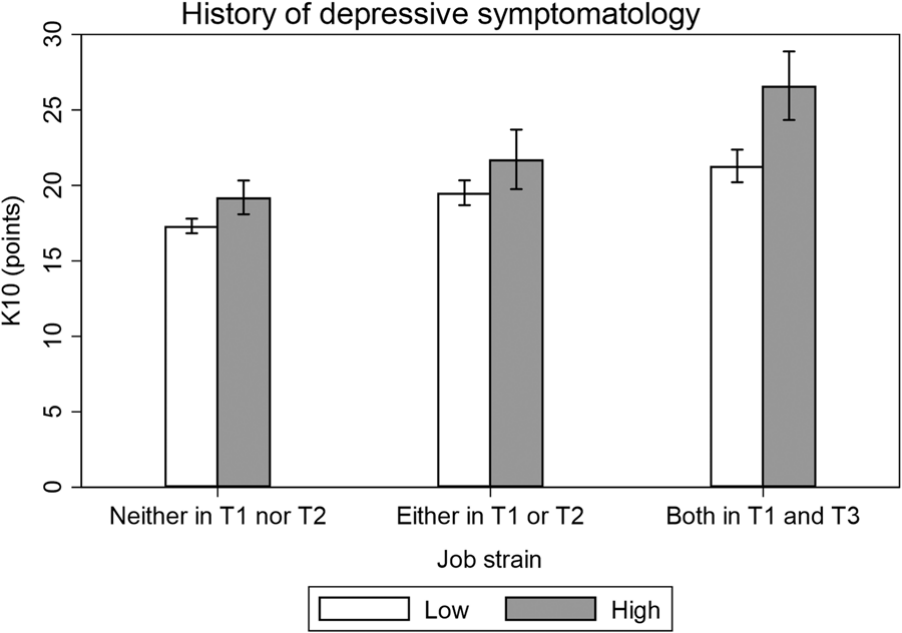
Estimated marginal mean (95% CI) effects of job strain on psychological distress (K10 by history of depressive symptomatology). Data are adjusted for gender, partner status, educational attainment, and socioeconomic status.

**Figure 4:**
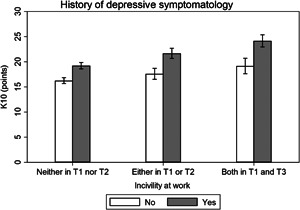
Estimated marginal mean (95% CI) effects of incivility at work on psychological distress (K10) by history of depressive symptomatology. Data are adjusted for gender, partner status, educational attainment, and socioeconomic status.

Effect modification analysis results are presented visually (Figures 1, 2, 3, 4) and with complementary detail in Table 2. For each of the stressors assessed, participants with no history of high depression symptoms consistently had the lowest distress levels among the unexposed, an intermediate level for those with depression history at one or the other timepoint, and the highest levels for those with depress at both time points in adolescence.

For low job control, the interaction may have been attributable to chance (Wald test *p* = 0.226). The difference in estimated marginal mean effects did not significantly differ between participants who reported high depressive symptomatology at both T1 and T2 (β [95% CI]: 1.80 [−0.41, 4.01], *p* = 0.110) or either T1 or T2 (0.88 [−0.94, 2.70], *p* = 0.341) compared with neither T1 nor T2 (Table 2 and Figure 2). For high job demands, the interaction was statistically significant (*p* = 0.015) and there was a difference in estimated marginal mean effects for participants who reported high depressive symptomatology at both T1 and T2 (β [95% CI]: 2.41 [0.22, 4.59], *p* = 0.031) compared with neither T1 nor T2, yet not for either T1 or T2 (−1.33 [−3.17, 0.51], *p* = 0.157) compared with neither T1 nor T2 (Table 2 and Figure 3); thus, supporting the presence of effect modification. For high job strain, the interaction was marginally significant (*p* = 0.053) and there was a difference in estimated marginal mean effects for participants who reported high depressive symptomatology at both T1 and T2 (*β* [95% CI]: 3.42 [0.64, 6.20], *p* = 0.016) compared with neither T1 nor T2, yet not for either T1 or T2 (0.32 [−2.14, 2.77], *p* = 0.800) compared with neither T1 nor T2 (Table 2; Figure 4); thus, there was evidence of effect modification. For incivility at work, the interaction was not statistically significant (*p* = 0.120), but the difference in estimated marginal mean effects may have differed between participants who reported high depressive symptomatology at both T1 and T2 (*β* [95% CI]: 2.02 [−0.12, 4.15], *p* = 0.064), though clearly not at T1 or T2 (1.10 [−0.61, 2.81], *p* = 0.208) compared with neither T1 nor T2 (Table 2; Figure 4); thus, there was suggestive evidence of effect modification.

## 4 DISCUSSION

Our results showed partial support for the hypothesis that a history of depression in adolescence predicts stronger associations between job stressor exposures and psychological distress among those employed in emergent adulthood. Though results for only two of the four analyses were able to formally exclude chance as a potential explanation, there was consistent pattern of approximately a doubling in the magnitude of association for participants with a history of high depression symptoms at two points in adolescence. Individuals exceeding the depression caseness threshold at both measured points in adolescence may have experienced more serious or sustained depression, whereas those who exceeded the threshold at only one of the two times were perhaps more mild or transient cases. This would suggest, on the basis of this study, that only more severe or on‐going depression could potentiate the impacts of subsequent exposure to job stressors in emergent adulthood.

In finding limited evidence of confounding of job stressor‐mental health associations by early life history of mental health problems, our results are consistent with two previous life course studies. In a UK 1958 birth cohort study, Stansfeld et al. found that childhood and early adulthood experiences of distress predicted job stressor exposures in mid‐adulthood (differential exposure), but did not explain associations between job stressor exposures and depression and anxiety disorder in midlife.^19^ Similarly, a New Zealand birth cohort study found that a history of psychiatric illness before labor market entry did not explain associations between job stressor exposures and incident depression or anxiety in young adulthood.^39^ Neither of these studies, however, investigated whether early life history of mental health problems modified the association between job stressors and mental health in adulthood (differential sensitivity).

With respect to our effect modification results, there are relatively few studies for direct comparison to ours. A Dutch cohort assessed the association between trajectories of mental health problems and work functioning among young workers, finding that those with persistent high levels of mental health problems had lower work functioning scores, and, in particular, had difficulties in meeting their work demands.^18^ Though answering a different research question, this study's finding is consistent with our finding that a history of adolescent depression modifies the association of job demands and distress. It is also notable in comparing our study to this one, that participants with a history of repeated high depression symptoms in our study had the highest mean K10 scores among unexposed groups. There are also other precedents for both differential exposure to job stressors in specific working population groups and differential sensitivity to the effects of job stressors on mental health.^20^, ^25^, ^40^, ^41^ For example, male workers report higher levels of job insecurity than females ^42^ and are also more sensitive to the effects of insecurity on mental health,^12^ both phenomena contributing to a disproportionate burden of job insecurity‐related ill‐mental health among males. The current novel finding suggesting differential sensitivity to the effect of some job stressors on mental health in emergent adult Victorian workers, however, may be context‐specific (with limited generalizability) and warrants replication.

Our study is limited in a number of ways. Our findings suggest that a purpose‐designed or more highly‐powered study using existing data is warranted to answer this important research question more definitively. Second, while the proposed effect modifiers were measured in Waves 1 and 2—prospectively in relation to the job stressor‐mental health relationship, the job stressor—mental health association was assessed cross‐sectionally at T3. Because job stressor and K10 were assessed in the same wave, there is the possibility of reverse causation. We would argue that this is not likely to explain the association between job stressors and K10 scores at T3 based on other studies in which we have shown that the relationship between job stressors and scaled measures of mental health is mostly contemporaneous in studies with one or more years between data collections.^12^, ^43^, ^44^ This contemporaneous association, we argue, represents a causally‐dominant stressor to mental health relationship, based on analyses of reversed and bidirectional associations,^43^, ^44^ and in the case of one stressor a strong prospective relationship between cumulative exposure and mental health.^12^ Other studies of job stressor exposure and mental health among young workers (15–30 years) have also shown contemporaneous exposure to be more strongly related with scaled measures of mental health than lagged by 1 or more years,^45^, ^46^ and a recent Dutch cohort showed that exposure to high demands or job strain at age 22 predicted depression and anxiety symptoms at age 29.^47^ Further, in a large population‐based Danish study, cumulative exposure to low job control was prospectively related to incident depression among workers aged 15–30.^48^ Taken together, this evidence suggests that job stressors affect scaled measures of mental health in the near term, and longer‐term cumulative exposures predict caseness of mental illness diagnoses.

In addition, our analyses are based on survey data, and we did not have objective measures with which to validate depression caseness. Because both job stressors and our outcome and effect modifiers were survey‐based, there is the potential for dependent misclassification (common method bias); this has been dealt with to the extent feasible in the studies supporting a causal relation between job stressors and mental health, as argued in the above paragraph. We used only the four job stressor measures that were available in this cohort, acknowledging there are many more job stressors that are negatively associated with mental health and whose association with mental health might be modified by a history of depression.^23^ A further limitation is that we have not considered the contributors to pre‐employment adolescent depression. However, mental health problems in children may be in part related to parents' working and employment conditions^49^; the intergenerational impacts of working and employment conditions warrant further research.

The limitations of this study are offset by various strengths, including the population‐representative sample, the high retention of study participants over three‐time points and a 12‐year time span, and the focus on adolescence and emergent adulthood as a critical period in the natural history of mental illness and socialization.^1^ Further, this study contributes to an emerging literature examining the impacts of adolescent mental health problems on the experience of work in adulthood.^17^, ^18^, ^47^

### 4.1 Potential relevance to policy and practice

The purpose of this study and line of research is to inform strategies for enabling and sustaining labor force participation among young workers with a history of mental health problems. Our findings suggest that psychosocial job quality may be an important—and modifiable—determinant of sustained employment among young workers with a history of high depressive symptoms. Supported employment and other functional recovery strategies for people with a history of mental illness can provide rehabilitative benefits and improved mental health.^50^, ^51^ Supported employment involves both assistance in obtaining employment and on‐going support on the job. The ability to assess the psychosocial quality of a specific job in the process of placement could help to avoid placement in jobs that would increase the job stressor‐associated risk of relapse or the incidence of new mental illnesses. This, however, is challenging to do without surveying all employees in the host workplace, which is usually infeasible. There is, however, some knowledge of the patterning of job stressor exposures across the working population, including in Australia.^52^ This information is provided by job‐exposure matrices, which provide estimates of typical job stressor exposures and exposure levels (intensities) by job title and sector across the working population. Job‐exposure matrices could be used to predict likely job stressor exposures in selecting or filtering placements in supported employment programs.

Once in a job, worker support could include periodic contact to monitor job stressor exposures at work. Existing instruments such as applied in this study and others^53^ could be readily adapted for this purpose, focusing for examples on experiences of incivility or bullying and social support at work, job control, job demands, and job strain. Where concerns arise, support could be provided directly to the worker and could, at least in theory, be provided back to the employer to inform primary prevention efforts (improvements in working conditions). In short, these strategies could plausibly improve the sustainability of employment for young workers with a history of mental illness.

It is also critical to consider that the majority of emergent adults working with an adolescent history of mental illness will not be in supported employment arrangements, and –based on our findings—some may be worse affected by job stressor exposures than those without such a history. This argues for stronger universal primary prevention strategies to reduce exposures to job stressors and to improve the psychosocial quality of work, as well as to enhance the development of workplace and other early intervention strategies that meet the necessary conditions of being timely, ethical, and nondiscriminatory.^54^ The related job stressor‐attributable burdens—even in the general working population—are substantial, and thus represent correspondingly substantial preventable mental illness burdens. Early estimates of job strain‐attributable depression, for example, were on the order 15% of prevalent depression in the general Australian working population,^55^ and subsequent estimates from Europe^56^, ^57^ and the UK have been comparable.^58^ Projecting from the results of the current analysis, should such estimates be doubled for emergent adults with a history of mental illness, this would suggest that there is considerable unrealized preventive potential in this area.

Finally, the COVID‐19 pandemic further emphasizes the importance of our findings. The pandemic has had significant adverse impacts on the mental health of young adults, including through disruption of education, increased isolation and loneliness, and bearing a disproportionate share of pandemic‐associated unemployment and underemployment.^8^, ^59^ There is also emerging evidence of deteriorating psychosocial working conditions for young workers,^60^ thus it is plausible that both differential exposure and differential susceptibility could be exacerbated by the COVID‐19 pandemic.

## 5 CONCLUSIONS

This study suggests that an adolescent history of high depression symptoms may potentiate the adverse impacts of job stressors on mental health in emergent adulthood. By our estimates, approximately 13% of emergent Australian adult workers have an adolescent history of high depression symptoms, thus representing an important societal concern. This study adds to the growing evidence that the prevention and control of exposure to job stressors should be considered as an important element of evolving strategies to protect and promote the mental health of emergent adults, both for those with and without a history of mental health problems.

## AUTHOR CONTRIBUTIONS

Anthony D. LaMontagne, Katrina Witt, and Allison Milner (AM, deceased) conceived of the study. Anthony D. LaMontagne, Katrina Witt, Allison Milner, and John W. Toumbourou were co‐investigators on the grant funding this research. Tracy Evans‐Whipp, John W. Toumbourou, and George Patton (deceased) provided access to the IYDS cohort and participated in final study design and analysis. Lay‐San Too and Patrick J. Owen conducted analyses with input and review of all co‐authors. Anthony D. LaMontagne wrote the paper, and all co‐authors have reviewed and approved the final version of the manuscript.

## CONFLICT OF INTEREST STATEMENT

The authors declare that there are no conflicts of interest.

## DISCLOSURE BY AJIM EDITOR OF RECORD

Paul A. Landsbergis declares that he has no conflict of interest in the review and publication decision regarding this article.

## ETHICS STATEMENT

Ethics approval was granted by Deakin University's Human Research Ethics Committee, protocol #2017‐124. Informed consent was obtained from all subjects and/or their legal guardian(s), and the study was carried out in accordance with the Declaration of Helsinki.

## Supporting information

Supporting information.

Supporting information.

Supporting information.

## Data Availability

The data that support the findings of this study are available on request from the last author: Prof John Toumbourou, Centre for Social and Early Emotional Development (SEED), Deakin University, Geelong, VIC, Australia; john.toumbourou@deakin.edu.au. The data are not publicly available due to privacy or ethical restrictions.
