# Supplementary figures and images for: Does adolescent depression modify the association between psychosocial job stressors and mental health in emergent adulthood?
Source: Am J Ind Med. 2023 Nov 4;67(1):44–54. doi: 10.1002/ajim.23547 (PMC10952472; doi:10.1002/ajim.23547)

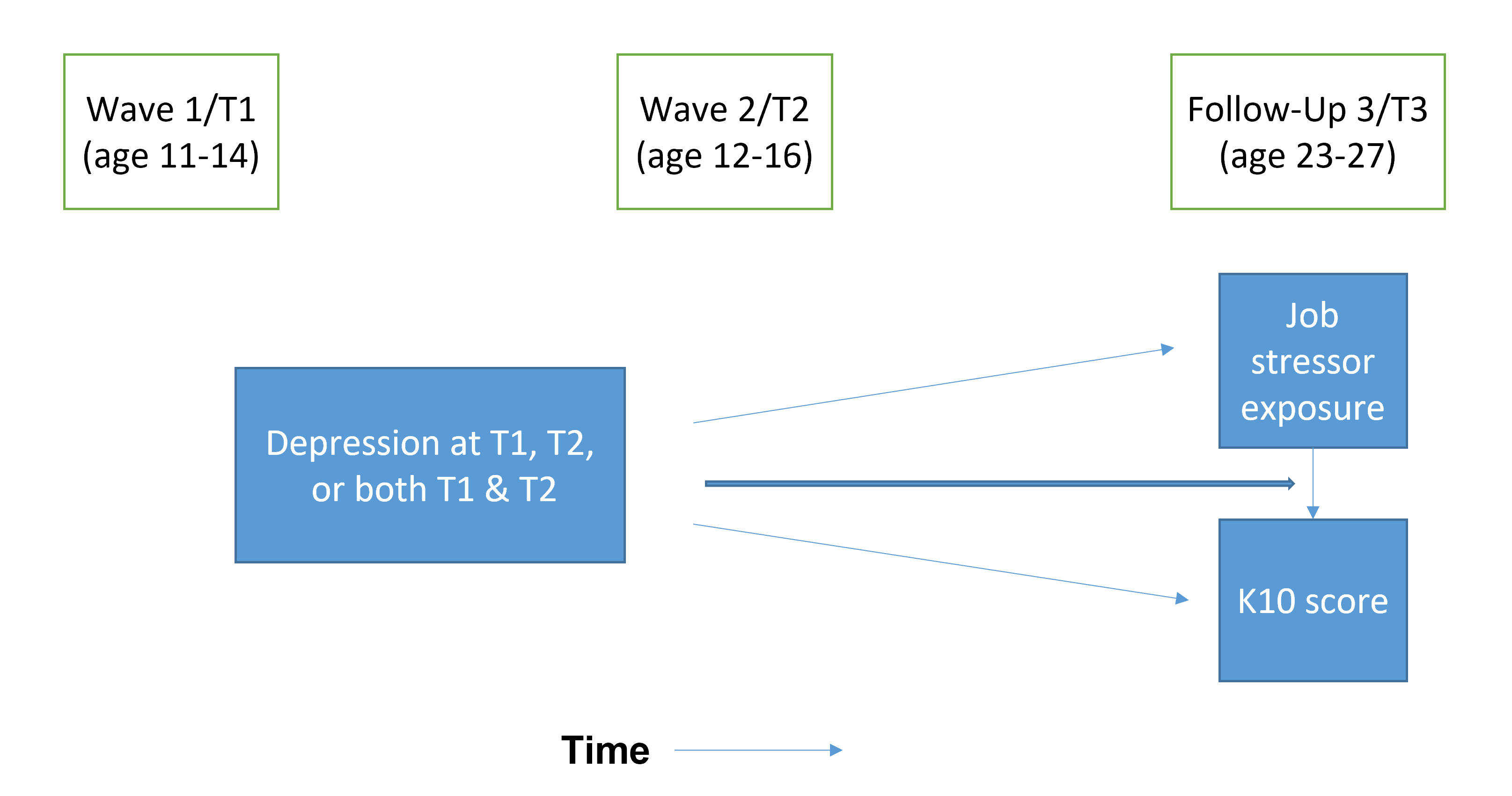

Supplement: Supplementary file 1 — Supporting information. [file AJIM-67-44-s002.tif]

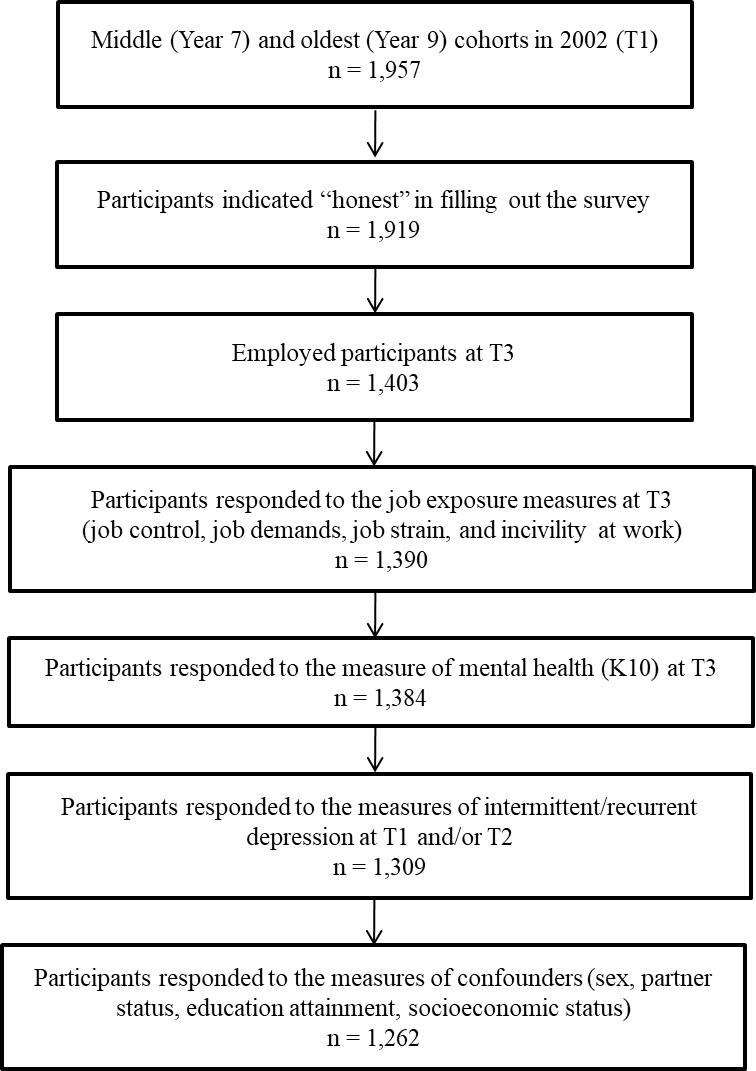

Supplement: Supplementary file 2 — Supporting information. [file AJIM-67-44-s003.tif]
