# Supplementary material for: Does adolescent depression modify the association between psychosocial job stressors and mental health in emergent adulthood?
Source: Am J Ind Med. 2023 Nov 4;67(1):44–54. doi: 10.1002/ajim.23547 (PMC10952472; doi:10.1002/ajim.23547)
Supplement: Supplementary file 3 — Supporting information. [file AJIM-67-44-s001.docx]

# Supplemental Table 1. Cohort participants working at T3: Comparison between participants included (n=1,262) and excluded (n=141) from analyses.

| Variable | Included | Excluded | P-value |
| --- | --- | --- | --- |
| **Psychological distress at T3, points** | 18.72 (6.74) | 18.24 (6.44) | 0.429 |
| **Low job control at T3, n (%)** | 501 (39.7) | 63 (46.3) | 0.135 |
| **High job demand at T3, n (%)** | 426 (33.8) | 48 (35.6) | 0.675 |
| **High job strain at T3, n (%)** | 199 (15.8) | 25 (18.2) | 0.472 |
| **Incivility at work at T3, n (%)** | 630 (49.9) | 71 (52.2) | 0.613 |
| **History of high depression symptoms, n (%)** |  |  |  |
| Neither T1 nor T2 | 815 (64.6) | 43 (67.2) | 0.384 |
| Either T1 or T2 | 279 (22.1) | 10 (15.6) |  |
| Both T1 and T2 | 168 (13.3) | 11 (17.2) |  |
| **Male at T3, n (%)** | 613 (48.6) | 67 (47.5) | 0.812 |
| **Having a partner at T3, n (%)** | 645 (51.1) | 60 (44.8) | 0.163 |
| **Education attainment at T3, n (%)** |  |  |  |
| Postgraduate/bachelor degree | 600 (47.5) | 62 (44.0) | 0.283 |
| Diploma/certificate | 530 (42.0) | 69 (48.9) |  |
| Year 12 | 97 (7.7) | 6 (4.3) |  |
| Year 11 or below | 35 (2.8) | 4 (2.8) |  |
| **Socioeconomic status at T1, points** | 1.97 (0.50) | 1.94 (0.56) | 0.517 |

Data are within-group mean (SD) or count (%) and between-group p-value. T1: time 1, T2: time 2, T3: time 3.

**Supplemental Table 2.** Relationships between individual job stressors and psychological distress (K10) at T3 in emergent adulthood.

| Variable | β (95%CI) | P-value |
| --- | --- | --- |
| **Low job control ^a^** |  |  |
| Model 1 | 1.31 (0.56, 2.07) | **0.001** |
| Model 2 | 1.46 (0.72, 2.19) | **<0.001** |
| History of high depression symptoms at both T1 and T2 ^b^ | 4.70 (3.61, 5.79) | **<0.001** |
| History of high depression symptoms at either T1 or T2 ^b^ | 2.29 (1.39, 3.18) | **<0.001** |
| **High job demand ^c^** |  |  |
| Model 1 | 2.43 (1.65, 3.20) | **<0.001** |
| Model 2 | 2.29 (1.54, 3.05) | **<0.001** |
| History of high depression symptoms at both T1 and T2 ^b^ | 4.49 (3.41, 5.57) | **<0.001** |
| History of high depression symptoms at either T1 or T2 ^b^ | 2.20 (1.31, 3.09) | **<0.001** |
| **High job strain ^d^** |  |  |
| Model 1 | 2.51 (1.51, 3.52) | **<0.001** |
| Model 2 | 2.48 (1.50, 3.45) | **<0.001** |
| History of high depression symptoms at both T1 and T2 ^b^ | 4.59 (3.50, 5.67) | **<0.001** |
| History of high depression symptoms at either T1 or T2 ^b^ | 2.25 (1.36, 3.14) | **<0.001** |
| **Incivility at work ^e^** |  |  |
| Model 1 | 3.80 (3.09, 4.51) | **<0.001** |
| Model 2 | 3.48 (2.78, 4.17) | **<0.001** |
| History of high depression symptoms at both T1 and T2 ^b^ | 4.08 (3.02, 5.14) | **<0.001** |
| History of high depression symptoms at either T1 or T2 ^b^ | 1.91 (1.04, 2.77) | **<0.001** |

Data are β (95%CI) and corresponding P-value. Model 1: Adjusted for gender, partner status and socioeconomic status. Model 2: Model 1 with adjustment for history of high depressive symptomatology as potential confounder. ^a^ compared to high job control; ^b^ compared to history of high depression symptoms at neither T1 nor T2; ^c^ compared to low job demand; ^d^ compared to low job strain; ^e^ compared to no incivility at work. T1: time 1, T2: time 2, T3: time 3.

# Supplemental Table 3. Relationships between job stressors and psychological distress (K10) at T3 in emergent adulthood, with mutual adjustment.

| Variable | β (95%CI) | P-value |
| --- | --- | --- |
| **Model 1** |  |  |
| High job strain ^a^ | 1.54 (0.57, 2.51) | **0.002** |
| Incivility at work ^b^ | 3.24 (2.53, 3.95) | **<0.001** |
| History of high depression symptoms at both T1 and T2 ^c^ | 4.09 (3.03, 5.14) | **<0.001** |
| History of high depression symptoms at either T1 or T2 ^c^ | 1.95 (1.09, 2.82) | **<0.001** |
| **Model 2** |  |  |
| Low job control ^d^ | 0.88 (0.17, 1.60) | **0.015** |
| High job demand ^e^ | 1.32 (0.56, 2.09) | **0.001** |
| Incivility at work ^b^ | 3.01 (2.28, 3.73) | **<0.001** |
| History of high depression symptoms at both T1 and T2 ^c^ | 4.11 (3.05, 5.16) | **<0.001** |
| History of high depression symptoms at either T1 or T2 ^c^ | 2.00 (1.13, 2.86) | **<0.001** |

Data are β (95%CI) and corresponding P-value. Both models adjusted for gender, partner status, socioeconomic status and history of high depressive symptomatology. ^a^ compared to low job strain; ^b^ compared to no incivility at work; ^c^ compared to history of high depression symptoms at neither T1 nor T2; ^d^ compared to high job control; ^e^ compared to low job demand. T1: time 1, T2: time 2, T3: time 3.
